# Ethyl pyruvate ameliorate inflammatory response of sinonasal mucosa by inhibiting HMGB1 in rats with acute rhinosinusitis

**DOI:** 10.1038/s41598-021-85785-3

**Published:** 2021-03-18

**Authors:** Xiang Liang, Yang Shen, Xiaowei Zhang, Guangxiang He, Guolin Tan

**Affiliations:** Department of Otolaryngology-Head Neck Surgery, Third Xiangya Hospital, Central South University, Changsha, 410013 Hunan China

**Keywords:** Medical research, Molecular medicine

## Abstract

High mobility group box 1 (HMGB1) has been known to involve in the pathogenesis of many inflammatory diseases. The aim of this study was to establish animal model of acute rhinosinusitis (ARS), and determine whether ethyl pyruvate (EP) attenuate inflammatory response of sinonasal mucosa by inhibiting HMGB1 in ARS animals. Thirty-six Sprague Dawley (SD) rat were used as follows: six normal controls without intervention (group 1); thirty rats were used for establishment of ARS rats model by nasal insertion of Merocel sponge, and model rats without any treatments (group 2), treated with nasal drops of sterile saline (group 3), 10 μl EP (group 4), and 20 μl EP (group 5), twice a day for 5 days, respectively. Bacterial culture was done regularly and the main bacterial strains were identified using matrix-assisted laser desorption/ionization time of flight mass spectrometry. HMGB1 expression in sinonasal mucosa was detected by immunohistochemistry and RT-PCR. Serum levels of HMGB1, IL-6, and TNF-α were determined by ELISA. Data from 29 of 36 rats that had completed research were analyzed. Bacterial colony formation unit (CFU) of nasal secretion was significantly higher in each group of ARS rats compared with controls (p < 0.001). ARS rats treated with EP had only slightly decreased CFU, but significantly attenuated inflammatory response of sinonasal mucosa and decreased HMGB1 expression compared to those treated with saline alone (p < 0.001). Serum levels of HMGB1, IL-6 and TNF-α were significantly higher in ARS rats compared to controls, and decreased by EP treatments (p < 0.001). Nasal sponge packing led to acute inflammatory response of nasal sinus in rats, and increased the expression of HMGB1, IL-6, and TNF-α. Nasal drops with EP could attenuate the inflammation of sinonasal mucosa through inhibiting the expression of HMGB1, IL-6 and TNF-α in ARS rats.

## Introduction

Acute rhinosinusitis (ARS) is defined by less than 12 weeks of purulent nasal drainage accompanied by nasal obstruction, loss of smell, and/or facial pain/pressure/fullness^1^. It is among the most common disorder encountered in ENT clinic, and one of the most common reasons for antibiotic prescriptions with antibiotics prescribed in 82–88% of patient visits^2^. However, antibiotics may not confer the benefit in lots of ARS, because the reported incidence of bacterial infection in ARS ranges approximately from 0.5 to 2%^1,2^. Overuse of antibiotics may lead to drug resistant. It deserves concern to reduce improper antibiotics use or apply non-antibiotic anti-inflammatory medicine in ARS patients.

Clinically, nasal mucosa inflammatory response of ARS involves fluid extravasation, mucus hyper-secretion, edema, and mucosal disruption. The inflammatory cascade of ARS involves type I inflammation-related cytokines, such as tumor necrosis factor(TNF)-α, β, interferon-γ, interleukin (IL)-1β, IL-6, which are associated with pathological process and outcomes of diseases^3^. Recently, a molecule, high mobility group box 1 (HMGB1), reportedly involved in the pathogenesis of many inflammatory diseases as a pro-inflammatory mediator^4^. There are evidences that an increased expression of HMGB1 was associated with the pathogenesis of chronic rhinosinusitis with/without nasal polyps, and secreted HMGB1 correlated with severity of inflammation in chronic rhinosinusitis^5^. However, the role it playing in ARS remains unclear.

Ethyl pyruvate (EP) is a derivative of pyruvic acid, and has anti-inflammatory effect through decreasing NF-kB-dependent signaling and down-regulating the secretion of pro-inflammatory cytokine, such as HMGB1^6^. Recent studies demonstrated that EP attenuated inflammatory response of murine allergic rhinitis by decreasing HMGB1 expression^7^. However, it remains to be elucidated whether EP also plays anti-inflammatory effects in ARS by inhibiting HMGB1 signals.

In the present study, a rat model of ARS was established by inserting the Merocel sponge into nasal cavity, and bacterial infectious inflammation was confirmed by bacterial culture of sinus secretion and pathological examination of sinonasal mucosa. The expression levels of HMGB1 were detected in the serum and sinonasal mucosa of ARS rats that intervened with or without EP treatments.

## Material and methods

### Animals

**Figure 1:**
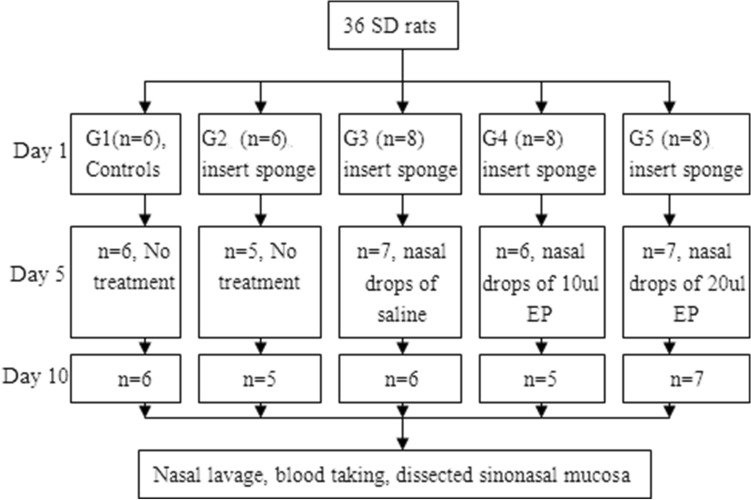
Group assignment. *G* group, *EP* ethyl pyrovate.

Thirty-six adult, male Sprague Dawley (SD) rats of clean grade were purchased from Experimental Animal Center of Central South University. The rats aged from seven to eight months and weighted from 250 to 280 g, and were feed with food and water all the time. The animals were divided into five groups (Fig. 1). Seven rats were unexpectedly dead during experimental period, and they were excluded in the study. All procedures were performed in strict accordance with Animal Studies Committee guidelines at Central South Unversity, Xiangya School of Medicine. The study was carried out in compliance with the ARRIVE guidelines ((https://arriveguidelines.org). All experimental protocols were approved by Ethics Committee of Third Xiangya Hospital, Central South University.

### Development of ARS rats’ model

The rat model of ARS was established using nasal packing of Merocel sponge as described previously^9^. Briefly, Merocel sponge was pruned into 2 × 3 × 8 mm^3^/each, and slightly inserted into right nasal cavity after achieving deep sedation anesthesia (3 ml/kg of 10% chloral hydrate intraperitoneal injection). Once awake and active, the animals were returned to the rodent care facility.

Before animal groups, in order to confirm that nasal packing can cause ARS, we first took five additional rats and inserted Merocel sponge into the right nostril of rats. It was found that the nasal sinuses were full of purulent secretions when the rats were killed and dissected five days later. Several strains of bacteria were identified from these secretions of sinus and nasal cavity by bacterial culture. It indicated that ARS can be developed through nasal packing for 5 days.

From day 5, the rats in each group were accepted different treatments for 5 days (Merocel sponge had not been withdrawn).

### Treatments

Six normal SD rats were selected as controls (group 1). ARS rats were just feed with food and water in group 2, and treated with nasal drops of 20 μl sterile normal saline, twice a day for 5 days, in group 3. Rats in group 4 and 5 were treated with nasal drops of 10 μl EP and 20 μl EP, respectively, twice a day, for 5 days (Fig. 1).

At day 10, a dose of 120 mg/kg pentobarbital sodium was given by intraperitoneal injection for terminal procedure of animals. Merocel sponge was removed from nasal cavity, and 500 μl sterilized normal saline was irrigated into nasal cavity, and lavage fluid was collected for bacteria analysis. Blood was extracted by eyeballs removal. Sinonasal mucosa was carefully dissected by surgical procedure, and each sample was cut into three parts for different experiments. All procedures were carried out following the rules of aseptic manipulation.

### Bacterial culture and strain identification

Each lavage sample was diluted as 1:100 using normal saline, and then took 10 μl diluted solution of nasal lavage fluid to spread onto agar medium culture dish. All dishes were kept in incubator at 37 °C for 24 h. The main strains were identified using matrix-assisted laser desorption/ionization time of flight (Maldi-Tof) mass spectrometry (Bruker Co., Germany). Colony formation unit (CFU) was used to estimate the number of bacterial colonies, and data was recorded as CFU per micro-liter of sinonasal lavage sample.

### Histology and immunohistochemistry assay

Routine histology was performed in sinonasal mucosa by paraffin-embedded and Hematoxylin and eosin (HE) staining in all samples. Immunostaining of HMGB1 was carried out in 29 samples of sinonasal mucosa as a method described previously^8^. Briefly, the 4-um-thick sections were deparaffinised. Antigen retrieval was performed in citrate buffer (0.01 M, pH 6.0) heated to boiling point for 5 min. Endogenous enzymes were inactivated by 3% hydrogen peroxide. HMGB1 immunostaining process was carried out by incubating slides with a rabbit poly-clonal HMGB1 antibody diluted at 1:500 (Abcam, Cambridge, UK) at 37 °C, then washed with PBS, and incubating with second antibody. Hematoxylin redyeing was performed on each slide. The expression of HMGB1 in sinonasal mucosa was scored using the semiquantitative system for both the percentage of positive cells and the staining intensity. The staining extent was scored as 0 (< 1%), 1 (1–25%), 2 (26–50%), 3 (51–74%) and 4 (≥ 75%), according to the percentage of the positive staining, whereas the staining intensity was evaluated as follows: no staining (score = 0), weak staining (score = 1), moderate staining (score = 2) and intense staining (score = 3). The final index was obtained by the sum of the intensity and percentage scores for each subgroup, ranging from 0 to 7.

### Quantitative determination of cytokines

The content of HMGB1, TNF-a, and IL-6 in serum were performed with enzyme-linked immunosorbent assay (ELISA) method. Human Quantikine ELISA kits (Shino-TEST Corporation, Japan) for HMGB1, IL-6, TNFα estimation were used. Sample concentrations were read from a calibration curve.

### Quantitative RT-PCR

Total RNA was extracted from sinonasal mucosa using an RNeasy Mini Kit (Qiagen, MD, USA). The reverse transcription reaction was carried out with 1 μg total RNA using 10 μM dNTP mix, 50 μM oligo (dT) primer, 5× First Standard Buffer, 0.1 M dithiothreitol, and SuperScript III Reverse Transcriptase according to the manufacturer’s protocols (Thermo Fisher Scientific, USA). The primers for HMGB1 were, forward, 5′-caaaatcaaaggcgagcatc-3′ and reverse, 5′-cagcttggcagctttcttct-3′; for β-actin were: 5′-acatccgtaaagacctctatgcc-3′ and 5′-tactcctgcttgctgatccac-3′. We performed quantitative RT-PCR using the ABI 7300 system (Applied Biosystems, Waltham, MA) under the following conditions: 95 °C for 10 min, followed by 40 cycles at 95 °C for 15 s and 60 °C for 50 s in 96-well plates in a final volume of 20 µl containing SYBR Green PCR Master Mix (Applied Biosystems). The relative quantity was calculated by the comparative threshold cycle (ΔΔCt) method using β-actin as the endogenous control.

### Statistical analysis

We performed statistical analysis using SPSS software (Ver., 17.0). Student’s *t*-test was utilized for HMGB1 positive cell score analysis and quantitative RT-PCR analysis. Values of *p* < 0.05 were considered significant.

## Results

### Bacterial strains of nasal lavage in ARS rats

**Figure 2:**
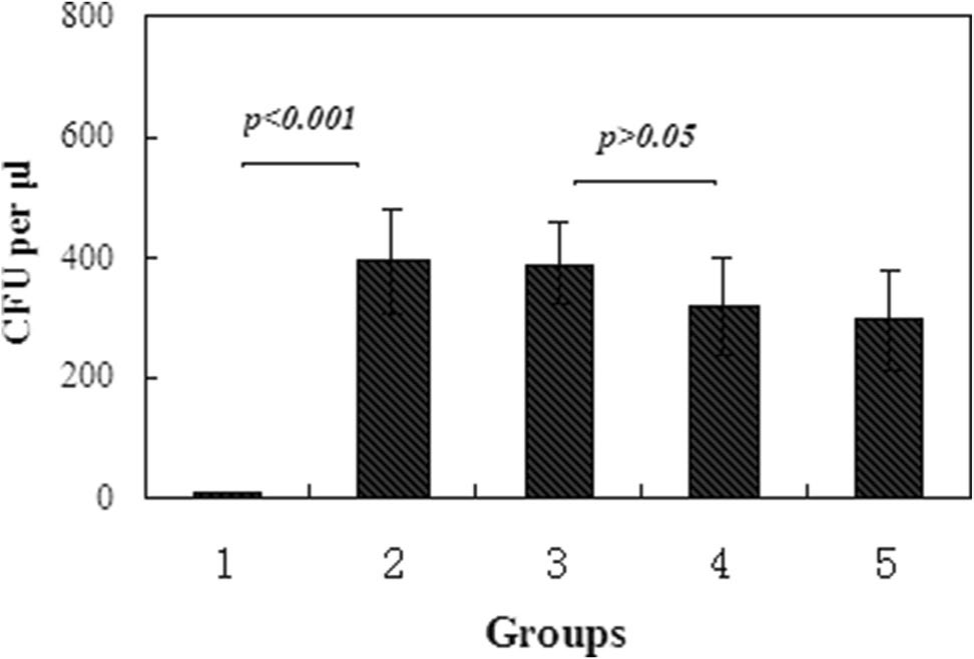
Colony formation unit (CFU) of bacteria was assayed as described in methods in five groups.

Bacterial culture of nasal lavage fluid in ARS rats treated with/without nasal drops of EP demonstrated that bacterial colonies were growing on each dish inoculated from group 1 to 5. The colony formation unit (CFU) was 9.5 ± 1.3/μl in group 1, 392.4 ± 86.8/μl in group 2, 389.5 ± 69.2/μl in group 3, 317.2 ± 79.5/μl in group 4, and 295.4 ± 83.4/μl in group 5 (Fig. 2). Compared with group 1, a significant increase of CFU value was found in group 2 to 5 (p < 0.001). However, The CFU value was only a slight decrease in group 4 and 5, compared with group 2 and 3 (p > 0.05). These findings suggested that Merocel packing could induce an ARS model that verified by following pathological examination. The main strains of bacteria in secretion of ARS rats were identified by Maldi-Tof mass spectrometry, and it included *Proteus mirabilis, Klebsiella pneumoniae, and Morganella morganii* in group 2 to 5. Pseudomonas agarici, a species of non-pathogen bacteria, was identified in group 1.

### Histologic HE staining of ARS rats

**Figure 3:**
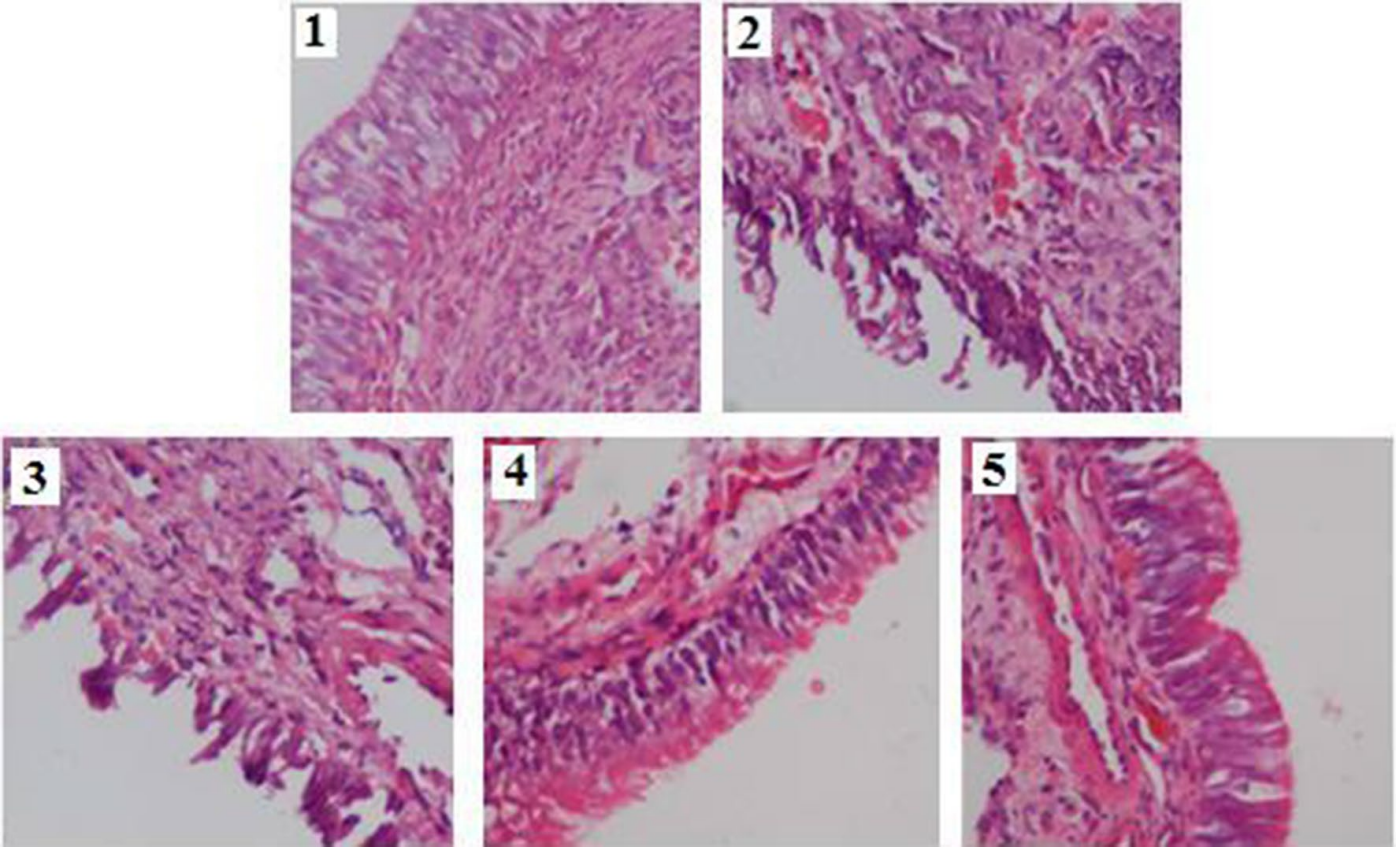
Representative HE staining of sinonasal mucosa from different groups. The number of photos represented group 1, 2, 3, 4 and 5, respectively.

**Figure 4:**
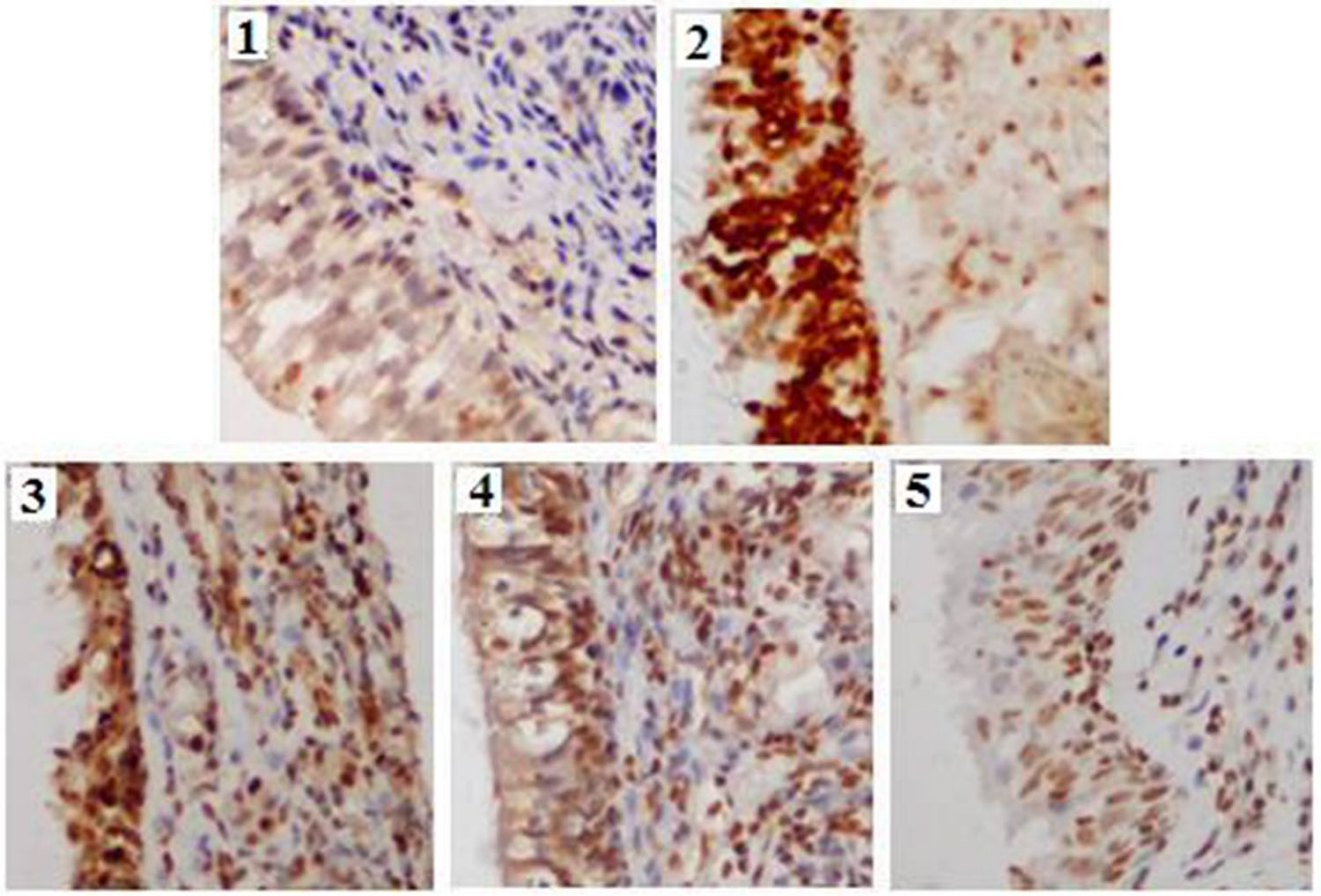
Representative immunostaining of HMGB1 in sinonasal mucosa from different groups. The number of photos represented group 1, 2, 3, 4 and 5, respectively.

**Figure 5:**
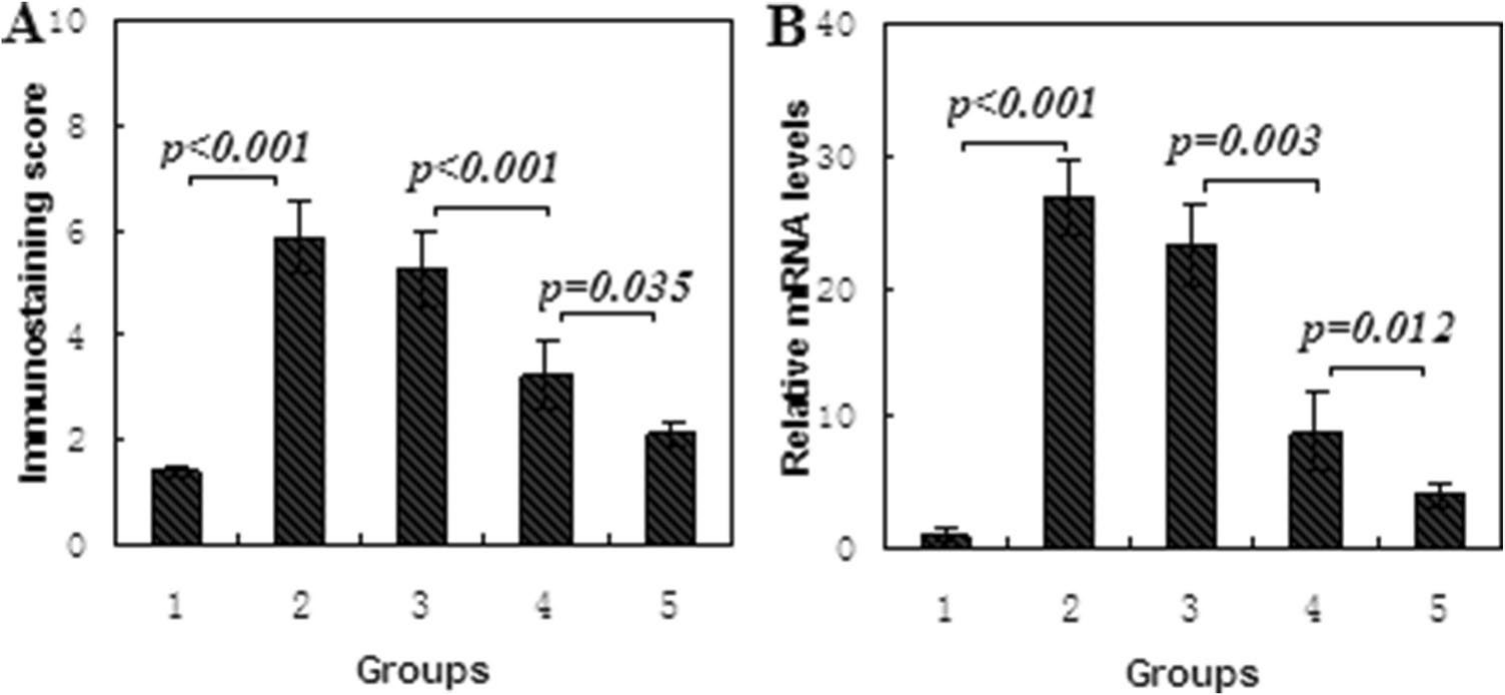
HMGB1 expression in sinonasal mucosa of rats from control, ARS and treatment groups. Data is presented as mean ± standard deviation. (**A**) Immunostaining score was assayed by immunohistochemistry; (**B**) mRNA level was detected by quantitative RT-PCR.

Histologic examination was performed by HE staining for rat’s sinonasal mucosa. Epithelial cilia desquamating, inflammatory cells infiltration, gland damage, vessel dilation and edema were observed in the sinonasal mucosa in ARS rats (Fig. 3-2), which was consistent with acute sinonasal sinusitis. From microscopic observation, the degree of inflammatory cell infiltration was attenuated in group 4 and 5 compared to group 3 (Figs. 3-3, 4, 5). However, no evidence of inflammatory clusters of sinonasal mucosa was found in control rats (Fig. 3-1).

### Expression of HMGB1 in ARS rats

The positive immuno-staining of HMGB1 was observed in sinonasal mucosa from both control (group 1) and ARS rats (group 2). The positive rate was quite low in epithelial cells and connective tissue, and only stained in nuclei in group 1 (Fig. 4-1), and its immunostaining score was 1.7 ± 0.23. The immuno-reactivity of HMGB1 was noted in epithelial cells, inflammatory cells and dilated vessels, and stained in both nuclei and cytoplasm in group 2 (Fig. 4-2), and the score was 5.8 ± 0.81, significantly higher than that in group 1 (*p* < 0.001, Fig. 4).

### HMGB1 expression attenuated by nasal drops of EP

In order to determine whether HMGB1 expression was modulated by treatment with EP, ARS rats in group 4, 5 were daily treated by EP solution drops, and HMGB1 expression was detected by immunohistochemistry and RT-PCR. The results showed: the score of HMGB1 immunostaining was 5.3 ± 0.72 in group 3, 3.2 ± 0.65 in group 4, and 2.4 ± 0.43 in group 5, and significantly lower in group 4, 5 than that in group 3 (*p* < 0.001, Figs. 4, 5A). Meanwhile, a 26-fold increase of HMGB1 mRNA expression was found in group 2 compared with group 1 (Fig. 5B, *p* < 0.001), and HMGB1 mRNA expression in group 5 was significantly lower than that in group 3 and 4 (*p* = 0.003 and *p* = 0.012, respectively). However, no significantly difference was found between group 2 and 3 (*p* = 0.083). These findings indicated that HMGB1 expression was also significantly attenuated by nasal drops of EP in sinonasal mucosa of ARS rats.

### Serum level of HMGB1, IL-6 and TNF-α in ARS rats

Serum concentrations of HMGB1, IL-6 and TNF-α were measured using enzyme-linked immunosorbent assays (ELISAs). The results obtained showed that serum levels of HMGB1, IL-6 and TNF-α were significantly higher in group 2 than that in group 1 (Table 1, *p* < 0.001), and nasal drops of EP caused these mediators significantly lower in group 4 and 5 compared with group 3 (*p* < 0.01, Table 1).

**Table 1:** Serum level of HMGB1, IL-6 and TNF-α was detected by ELISA in five different groups (M ± S).

Groups^a^	HMGB1 (pg/ml)	IL-6 (pg/ml)	TNF-a (pg/ml)
1 (n = 6)	519.5 ± 57.6	38.5 ± 4.5	8.2 ± 1.9
2 (n = 5)	2841.5 ± 41.7^b^	88.5 ± 11.4^c^	28.3 ± 6.7^b^
3 (n = 6)	2732.6 ± 87.9	81.0 ± 12.7	27.5 ± 6.1
4 (n = 5)	1054.5 ± 107.1^c^	64.5 ± 10.7^c^	13.1 ± 2.3^c^
5 (n = 7)	647.4 ± 91.6^c^	50.8 ± 6.72^c^	10.1 ± 1.8^c^

Mean ± standard deviation.

^a^Group 1, controls; group 2, ARS group without treatments; Group 3, ARS rats treated by normal saline; Group 4, ARS rats were daily treated by 10 ul ethyl pyrovate, twice a day. Group 5, treated by 20ul ethyl pyrovate, twice a day.

^b^*p* < 0.001 when compared with group 1.

^c^*p* < 0.05 compared with group 3.

## Discussion

Acute rhinosinusitis is a common inflammatory disease in Rhinologic clinic. Several animal models of ARS were developed to investigate its mechanism^9^. A study demonstrated that nasal sponge insertion could induce ARS mouse model, but inflammatory response was less severe than that nasal sponge insertion impregnated with *Staphylococcus aureus* suspension^9^. In our present study, data showed that nasal sponge packing could cause sinonasal bacterial infection in all rats, and main bacterial strains included *Proteus mirabilis, Klebsiella pneumoniae, and Morganella morgani*, which were conditional pathogens. Although the strains of bacteria isolated from these rats were not the typical ones found in ARS patients in human, the pathological features of acute rhinosinusitis were observed by HE staining in these packing-induced ARS rats, such as epithelial cilia desquamating, inflammatory cells infiltration, gland damage, vessel dilation and edema. It indicates that nasal sponge packing can lead to ARS in rats, and it is similar with clinical results of nasal packing after nasal surgery^10^. It needs to be intervened with some treatments.

Cytokines, as key regulators of inflammation, plays an important role in the pathophysiology of ARS. Series of up-regulated cytokines were detected in acute viral rhinitis, and included IL-1β, IL-6, IL-7, IL-17, IFN-γ, TNF-α, IL-8, G-CSF, GM-CSF^11^. Bacterial unmethylated CpG administration into the human nose leads to accumulation of Th1-related cytokines IL-1β, IL-6, and IL-8^12^. Increased values for IL-2, IL-4, IL-10, IL-12, IL-13, TNF-α, and IFN were detected in nasal fluids from ARS patients compared to chronic rhinosinusitis^13^. However, the values for IL-12 and IL-4 were not increased in murine model of acute bacterial rhinosinusitis^14^. The defined cytokine response remains largely unclear in ARS. Our data demonstrated that serum level of IL-6 and TNF-α was significantly increased in nasal packing-induced ARS rats, which were mainly Th1-related cytokines, and consistent with pathogenic mechanism of ARS.

HMGB1 is an evolutionarily highly conserved protein as a chromatin-binding molecule and named for its high electrophoretic mobility on polyacrylamide gels^4^, and it is widely expressed in all nucleated mammalian cells. HMGB1 binds to Toll-like receptor 4 (TLR-4) and the receptor for advanced glycation end products (RAGE), activating the NF-κB signaling pathway and inducing the release of pro-inflammatory mediators, cytokines and chemokines^15^. It has been shown to play a role in the pathogenesis of several inflammatory diseases like hepatitis, arthritis, stroke, liver and kidney ischemia, sepsis, rheumatoid arthritis, systemic lupus erithematosus^16^. Recently, studies demonstrated that HMGB1 was highly expressed in epithelial cells and inflammatory cells of patients with chronic rhinosinusitis and allergic rhinitis, and patients with severe symptoms have the highest serum levels and the highest extracellular expression of HMGB1^5,17^. Shimizu, et al. reported that nasal secretions from chronic rhinosinusitis contain higher amounts of HMGB1, and TNF-α stimulated the production and secretion of HMGB1, and HMGB1 stimulated the production and secretion of IL-6 and IL-8 by cultured nasal epithelial cells. Meantime, anti-TLR-4 antibody significantly decreased HMGB1-induced secretion of IL-6 and IL-8^18^. In our present study, a higher expression of HMGB1 was found in both nuclei and cytoplasm of epithelial cells, inflammation cells and vessels in sinonasal mucosa of packing-induced ARS rats. The immunostaining score of HMGB1 was significantly higher in ARS group than that in control group. Simultaneously, serum contents of HMGB1, TNF-α, and IL-6 were also significantly increased in ARS rats. These results suggested that HMGB1 may play a crucial role in pathogenesis of ARS through intergenic regulation with TNF-α and IL-6, and could be a target for treatment.

Although antibiotics are often used for control the bacterial infection in ARS, antimicrobial resistance is a global problem hindering treatment of bacterial infections. To avoid the antibiotic resistance, developing non-antibiotic anti-inflammatory medicine for bacterial inflammation could be a reasonable strategy. Glycyrrhetic acid and hyaluronic acid, as non-antibiotics medicine, have been reported to be effective for ARS^19^. Recently, anti-inflammatory effect of ethyl pyruvate (EP) has been mostly noticed by scientists. Treatment with EP is able to ameliorate systemic inflammation and multiple organ dysfunctions in multiple animal models, such as sepsis, acute pancreatitis, alcoholic liver injury, acute respiratory distress syndrome, acute viral myocarditis, and acute kidney injury^6^. Recent data showed that EP attenuated the inflammatory response of murine allergic rhinitis and asthma^7^. The biomedical mechanisms responsible for anti-inflammatory effect include inhibition of pro-inflammatory transcription factor, NF-kB, as well as expression of several pro-inflammatory proteins, such as TNF-α, IL-6, cyclooxygenase-2, and inducible nitric oxide synthase^20^. Administration of EP attenuated inflammatory response and reduced HMGB1 release in mice with sepsis or murine allergic rhinitis^7^. In our current study, the data demonstrated that nasal drops of EP (20 ul, twice a day) significantly decreased scores of HMGB1 immunostaining and mRNA expression, and lowered the serum content of HMGB1, TNF-α, and IL-6 in ARS rats. The pathological inflammatory response was also attenuated in sinonasal mucosa of ARS rats. However, the CFU of nasal secretions was not significantly changed by EP treatment in nasal packing-induced ARS rats. These findings suggest that EP has a strong anti-inflammatory effect through inhibiting expression of HMGB1, TNF-α and IL-6, rather than inhibiting microbial growth.

Although this study demonstrated that EP inhibited inflammatory response of sinonasal mucosa in ARS rats, the optimal EP concentration and appropriate time course of treatment have not been explored, and its detailed mechanism is also far from clarified. Therefore, it needs further studies. It is not clear whether nasal irrigation of EP solution could be benefit to ARS patients in attenuating inflammatory response of nasal sinus, and clinical trials are necessary for the role of EP on patients with ARS.

## Conclusion

Nasal packing caused acute inflammatory response of nasal sinus in rats, and treatment with EP could attenuate the inflammatory response by inhibiting the HMGB1 expression in serum and nasal mucosa.
